# The contribution of social participation to differences in life expectancy and healthy years among the older population: A comparison between Chile, Costa Rica and Spain

**DOI:** 10.1371/journal.pone.0248179

**Published:** 2021-03-12

**Authors:** Sarahí Rueda-Salazar, Jeroen Spijker, Daniel Devolder, Cecilia Albala

**Affiliations:** 1 Centre d’Estudis Demogràfics (CED), Autonomous University of Barcelona (UAB), Barcelona, Spain; 2 Unidad de Nutrición Pública, Instituto de la Nutrición y Tecnología de los Alimentos (INTA), Universidad de Chile, Santiago, Chile; University of Botswana, BOTSWANA

## Abstract

We study the health trajectories of the population aged over 60, comparing between one European and two Latin American countries (Spain, Chile and Costa Rica) which have similar longevity patterns. Our focus is on functional limitation and mortality risks, considering differences by gender, education and social participation. Data come from national panel surveys (EPS, CRELES, SHARE). Multistate modelling is used to estimate transition probabilities between two health states: healthy to unhealthy, unhealthy to healthy as well as the transition to death from healthy or unhealthy states, to estimate the duration of stay in a specific state (computing healthy and unhealthy life expectancies) and the effect of the selected covariates. Results show that older Costa Ricans have the smallest gender gap in life expectancy but women have a lower healthy life expectancy compared to those in Chile and Spain. Participation in social activities leads to higher healthy life expectancy among the elderly in Costa Rica and Spain, whilst there were no relevant educational differences observed in longevity in the analysed countries. To conclude: despite the different patterns observed in health transitions and survival across the three countries, social participation is associated with greater health and longevity among people of old age, with little effect coming from educational attainment. Public policies should therefore be aimed at reducing unhealthy life years and dependency at advanced ages by promoting more engagement in social activities, especially among vulnerable groups who are more likely to experience impairment from a younger age.

## Introduction

Ageing in Latin America and the Caribbean (LAC) is a relatively recent phenomenon in contrast to Europe. However, in recent decades this region has witnessed the most rapid increase in the proportion of older population compared to other regions and other periods in history [1–3]; in the period 2000–2020 it increased by 57% (from 8.3% to 13.0%), with a growth similar to Asia, while European countries only recorded an average increase of about 25% [1]. The speed of the ageing process in LAC is largely due to the combined effect of significant declines in mortality at advanced ages and even sharper decreases in fertility levels since the 1960s, both of which have intensified over the last 2–3 decades [1,4,5].

The countries with the largest proportion of older people (60+) in LAC in 2020 are Cuba (21.3%), Uruguay (20.2%), Chile (17.4%), Argentina (15.5%) and Costa Rica (15%) [1]. The population of these countries also have the longest mean life span in the region [6], with life expectancies (LEs) ranging from 80–82 years for women and 73–79 years for men. However, as we will argue later, the level of LE does not exactly provide sufficient information on the health status and quality of life of the older adults.

Nowadays, the health of older people is represented by the preservation of their physical and mental capacities needed to function in their immediate environment ─which allows for well-being in old age─rather than by the absence of disease or experimenting bad health conditions [7]. Consequently, the ability of the older people to live independently, understood as the possibility for a person to carry out daily activities without receiving supervision or help from others [8], is the main objective for an active and healthy ageing [9].

Various studies indicate that the population of LAC countries that reached sixty years of age in the 2000s has benefited from the deployment of innovations in medicine in childhood and infancy which took place after 1945 [10]. Hence, and in comparison with previous birth cohorts, their better survival is mainly due to improvements in medical treatment and less exposure to contagious diseases, rather than to improvements in living conditions. However, at the same time, fragile economies, rising poverty levels, expanding economic social and economic inequalities and contracting access to collective financed services and resources in LAC [11] are likely to play a part in lower healthy LEs at older ages than those observed in European countries that have a strong welfare state. For instance, at age 60, the average HLE in LAC was 16.1 years in 2019. This is 2.5 years lower than the average for Western Europe. The highest HLE in LAC is recorded in Costa Rica (18.7 years), which is just above the Western European average, but below that of Spain (18.8 years), Sweden (18.9 years), Switzerland (19.5 years) and France (19.7). Chile also has one of the highest HLE among LAC (18.0 years at age 60), but is lower than most Western European countries [6]. Latin American health studies relate their worse health conditions among elderly to poor conditions experienced early in life [12]. Moreover, it is expected that disability at older ages will actually increase in future years as chances of surviving to old age have also improved [13]. Some comparative studies between European countries and Latin America have shown that differences in levels of functional limitations at older ages are also related to differences in general economic welfare, as well as accessibility to and quality of public services, in particular health care and primary attention [14] which are better developed and more effective in Western Europe. This implies a growing demand for health care and attention needs at older ages in the case of LAC [15,16]. In the case of Southern European countries, including Spain, this is also an issue [17–19], as public institutions do not have enough capacity to manage the provision of care for the elderly, reason why the family is still the fundamental basis for elderly support and care there. In contrast, in Nordic and Western European countries the State is the main provider of such care [20–23].

In LAC, family ties have created a compensatory system of intergenerational support networks within extended family households ─an expansion of the nuclear family─ [4,11,16,19,24–29], as is the case in Chile and Costa Rica. While nuclear families are more common in Spain, its average number of household members is higher than in Northern and Western European countries [30], where the partner (women in most cases) represents the largest and principal caregiver [17,31–34].

The timing and duration of the demographic transition in each of the Latin American countries [35] have direct consequences on the availability of relatives and on the support network of the older population [19]. Consequently, the countries with the earliest decline in their fertility rates will be those with the highest proportions of elderly people living alone and the lowest proportions of living with children, as is the case of Uruguay and Argentina [15].

In this context, social participation, which includes volunteering, participating in civil or religious organisations, doing sports, etc., emerges as part of successful strategies for preserving independence when older workers transition from labour market exit to retirement [36] and consequently contributes in reducing the costs of long-term care for those in the fourth age [37]. Social participation is considered an important tool to promote the comprehensive well-being of this population group in the guidelines for active aging by the WHO [38]. Studies indicate that social participation not only reduces social exclusion [39] but also have various benefits for the wellbeing of older people in different countries [37,40,41]. In general, they broadly favour two health domains: cognitive processes (increasing cognitive functioning) and prevention of risk factors. The former is associated with psychological objectives focused on emotions such as reducing anxiety, increasing life satisfaction and wellbeing. The latter is aimed at stimulating healthier behaviours in terms of diet, alcohol consumption and smoking [9].

However, studies on elderly health trends in LAC are still scarce [12,42–50], and even more so between-country comparisons [51–53]. Most studies are descriptive [26], based on the Survey on Health and Well-Being of Elders (SABE) [11]), conducted in seven cities in LAC. This survey is one of the few conducted in the region for studying elderly health conditions since the turn of the century. It was designed to be cross-sectional, although in Santiago de Chile, Sao Paulo and Mexico City there were longitudinal follow-ups.

Most of the analyses on health conditions in LAC are based on the use of prevalence rates for the estimation of Healthy Life Expectancies (HLEs) by means of the Sullivan method [47,54,55] with the exception of the study by Moreno, Albala and Lera et al. [56] who use incidence rates to estimate multiple state models. As we also use longitudinal panel survey data, the same method is applied in this study. Incidence rates are more robust than prevalence rates because they capture changes in health status over the life cycle (i.e. across age) and therefore, HLE estimations are closer to the health dynamics experienced by the population [57–60].

The present study analyses the functional health trajectories of the elderly in order to compare recent health trends of the population over 60 years of age between three countries: Chile and Costa Rica ─known in Latin America for their high LE─ and one European country, namely Spain. There are two main reasons for their selection. Firstly, a practical one, as excellent national-level longitudinal data exist for all three countries. Secondly, the three countries are similar in terms of life expectancy, elderly educational levels and income. For instance, in 2019, LE at age 60 in Chile was 22.4 years for men and 25.9 years for women, in Costa Rica 23.6 and 26.4 years, and in Spain 22.6 and 26.7 years respectively [6], with gaps between TLE (Total Life Expectancy) and HLE of 5.9 years in Chile and 6.3 years in Costa Rica and 6.2 years in Spain (both sexes combined). However, at the same time, their health care systems are quite different, which, in the context of the obtained results, is an issue we briefly reflect upon in the discussion.

More specifically, the study explores the differences between countries in the level of transition probabilities between two health states: healthy to unhealthy, unhealthy to healthy, as well as the transition (from the unhealthy and healthy state) to death. This allows computing the expected duration of staying in a specific state, i.e. the healthy and unhealthy life expectancy at age 60. We also consider differences according to gender, educational attainment and social participation. The analysis focuses on the contribution of these variables to the risk of each health trajectory as they are considered to play a role in the conservation of functional capacities at advanced ages and thereby in the reduction of dependency and the needs for long term health care.

## 2. Data and method

Two waves of three longitudinal panel-type surveys are used for each country (see Table 1): Chile’s Social Protection Survey "EPS" [61] (years 2004 and 2006), Costa Rica’s Longevity and Healthy Aging Study "CRELES" [62] (years 2005 and 2007) and for Spain the "Surveys of Health, Ageing and Retirement in Europe” (SHARE) [63] (years 2004/05 and 2007). Although each survey has additional waves, we only analysed the interval between the first two waves as this was the only way to ensure that countries could be compared and that the issue of attrition was minimal. However, all waves were analysed and the results are provided in the Supplementary material.

**Table 1:** Chile, Costa Rica and Spain: Population (60+) Final sample distribution by age, educational level and social participation.

Demographic Indicators	Chile (EPS)	%	Costa Rica (CRELES)	%	Spain (SHARE)	%
Population 60+	3,344	100.00%	1,970	100.00%	1,317	100.00%
Gender						
Male	1,681	50.27%	913	46.35%	579	43.96%
Female	1,663	49.73%	1,057	53.65%	738	56.04%
Age groups (Male)						
[60,65)	540	32.12%	121	13.25%	132	22.80%
[65,70)	406	24.15%	191	20.92%	130	22.45%
[70,75)	345	20.52%	193	21.14%	144	24.87%
[75,80)	201	11.96%	180	19.72%	98	16.93%
[80,85)	111	6.60%	126	13.80%	45	7.77%
[85,90)	55	3.27%	65	7.12%	22	3.80%
[90,+)	23	1.37%	37	4.05%	8	1.38%
Age groups (Female)						
[60,65)	472	28.38%	162	15.33%	154	20.87%
[65,70)	394	23.69%	233	22.04%	152	20.60%
[70,75)	292	17.56%	207	19.58%	154	20.87%
[75,80)	246	14.79%	168	15.89%	130	17.62%
[80,85)	142	8.54%	151	14.29%	90	12.20%
[85,90)	79	4.75%	103	9.74%	43	5.83%
[90,+)	38	2.29%	33	3.12%	15	2.03%
Educational level						
No studies	359	10.74%	294	14.92%	372	28.25%
Primary	1,875	56.07%	1,367	69.39%	668	50.72%
Secondary	906	27.09%	176	8.93%	213	16.17%
Tertiary	176	5.26%	133	6.75%	55	4.18%
Others	-		-		8	0.61%
N/R	28	0.84%	-		1	0.08%
Social Participation						
With participation	1,076	32.18%	1,146	58.17%	307	23.31%
With no participation	2,265	67.73%	815	41.37%	992	75.32%
N/A^1^	3	0.09%	9	0.46%	18	1.37%

### Chile: Longitudinal Social Protection Survey (EPS)

The nationally-representative EPS survey was first conducted by the “sub-secretaria de Protección Social” in 2004 aimed to provide information on the socioeconomic characteristics, family history, household conditions, work history, the social protection system (pensions) and health conditions of the population aged 18 and over [64]. 3,562 respondents aged 60+ participated in the baseline sample. Of those, 3,344 also participated in the 2007 wave (see S1 Fig). And, overall, the survey did not use proxy respondents in any of their waves.

### Costa Rica Study on Longevity and Healthy Aging (CRELES)

The CRELES survey is the only source of longitudinal information on older people (60+) in Costa Rica. It is conducted by the Central American Population Centre (CCP) and the Health Research Institute (INISA) of the University of Costa Rica with the collaboration of other national institutes. This survey collects information on household conditions, socioeconomic characteristics, lifestyles, and specific health information (i.e. self-perceived health, physical examinations: blood pressure, anthropometry, mini-mental test, blood and urine collection which allows the study of a wide variety of bio-markers). We used data from the 2005 (baseline) and 2007 rounds without including new interviewed individuals after the baseline [75]. In the 2005 baseline sample, there were 2,827 respondents aged 60+, out of whom 2,631 also participated in the first follow-up (see S2 Fig). For the purpose of our analysis, we also excluded 661 respondents from the baseline sample who required a proxy respondent, which left us with 1970 cases. Proxies are usually employed when the main respondent is not able to do the interview because of health reasons. Taking a closer look at these respondents, their age profile showed that 75% were aged 80+, which is not surprising given the high oversampling of older people at baseline. Removing these cases from the sample therefore made the age-distribution more comparable with that of Chile and Spain. Moreover, the life expectancy at age 60 of these respondents was approximately 10 years less than that of respondents who partook the interview without the help of someone else. Including these cases would have biased the results too much (see also the Discussion).

### Health, Ageing and Retirement Survey of Europe (SHARE)

SHARE is a longitudinal (panel-type) survey that collects information on health, socio-economic status, employment trajectories, family (including the partner of the selected persons regardless of their age) and social networks of 140,000 individuals aged 50 and over in 27 European countries and Israel. Currently, information is available for 7 rounds, covering the period 2004–2017 [65]. However, although data is available for more recent rounds, only those respondents from the Spanish sample aged 60 and over who participated in the 2004 and 2007 waves were selected to enable the results for Spain to be compared with those of Chile and Costa Rica. From the 1,554 individuals who were interviewed at baseline, 1,372 persons also participated at the first follow-up, 1317 of whom without a proxy respondent and with a known health state (see S3 Fig). Respondents with a proxy respondent were removed to be consistent with the sample methodology applied to Costa Rica.

### Measurements

The health status (dependent variable) of the population under study is estimated through the self-reporting of functional capacity to perform four Basic Activities of Daily Living (ADLs), namely: bathing, walking across a room, getting in or out of bed, and eating (see S2 Table). Therefore, the individual who did not report any of the four ADLs described above is considered to be healthy. These criteria are also used in the estimation of healthy and unhealthy years of life. We choose this indicator as it can be constructed from the data for all three countries under study and, as we explain in the discussion, the obtained results are compatibles with alternative indicators.

For the identification of the dead state, required for the calculation of transition probabilities between each health state and death (see method section), we were able to obtain the exact date of the occurrence. In the case of Chile, the information on deaths came from civil registries that were added to the survey, while in the Costa Rican and Spanish surveys, the retrospective information on the dates of deaths in the household was reported by a household member [66,67].

The main covariables in our analysis are: education and social participation. The questions on social participation vary according to survey (see S3 Table) but we were able to dichotomise the responses to whether or not the respondent was participating in any activity during the last six months). The education variable was also dichotomised into primary or less and secondary or more. Finally, sex and age are control variables.

### Method

The methodology used is based on the estimation of multi-state transition models. These models are used in survival analysis and allow the calculation of transition probabilities between health states by means of a stochastic process. These models consider changes in health status on the life cycle of individuals exposed to current morbidity and mortality conditions. This means that they capture health dynamics of the population between surveys waves through incidence rates. Hence, these are calculated transition probabilities between health states at a specific time period delineated by two ages ***p_ij_***(***x,y***) (probability that a person in state ***i*** at age ***x*** will be in state ***j*** at age ***y***). The use of these models also allows us to estimate reversible transitions [68], something which is not possible when using prevalence rates (e.g. Sullivan method).

Multi-state models also allow to simultaneously explore the effect of variables on transitions between states and, finally, to estimate the duration of staying in a specific state. In the context of these models, all the LEs that we report here correspond to the expected duration of time regardless of the initial state occupied (Marginal Life Expectancy).

In this study we used an "Illness-death model with recovery" with three states: two transitory states: “healthy” and “unhealthy”, and a terminal (absorbing) state that corresponds to “death”. This is a mixed model [69] where the first two states are censored in the interval of two waves, that is, the exact moment when the transition between the health states occurred is not known, but the time interval between transitions is known, which corresponds to the date of the surveys.

The "healthy" and "unhealthy" states are recurrent as individuals may enter and exit them as many times as they are observed, and the “death” state only allows, by definition, entry into this state, once.

We used the R package "msm", version 1.6.7 [70] for the estimation of the “multistate survival models” to derive the hazard rate or instantaneous probability [71] of the transitions between states and for computing the hazard ratios of the explanatory variables (see S3 Table). These models include age as a continuous dependent variable, which means that transition probabilities between states increases or decreases log-linearly with age, following a Gompertz function. In this case, the probability function is estimated using a constant piecewise approximation conditioned by age [72], which means that the instantaneous probability of transition between states is constant within each age interval.

However, the “msm” package only allows estimating life expectancies estimates based on an exponential function, using age-constant rates [72]. We therefore used the R-package "elect", version 0.2 [73] which can use the model based on the Gompertz function to derive so-called “marginal” life expectancies [74]. These are the expected times spent in healthy or unhealthy states, regardless of the initial state of occupancy, as well as the state-specific life expectancy that refers to the expected occupancy time in each state.

Specifically, life expectancies are estimated based on numerical integration, which also provides an extrapolation of the previously calculated multistate survival model beyond the age range in the data. Additionally, the multinomial logistic regression model is applied using the state distributions at baseline (first observation) to estimate the effects of the time-independent covariates (age, education and social participation) on life expectancy and healthy life expectancy (see Van den Hout [74], section 7.3). Confidence intervals for the estimated values of the life expectancies were also calculated from 500 repeated simulations based on the asymptotic properties of the maximum likelihood estimator applied for the multi-state model [72,74,75], thereby sampling the stochastic process.

In this study we limited ourselves to estimate the total marginal life expectancy (TLE), the marginal life expectancy in healthy state (HLE) and the marginal life expectancy in unhealthy state (ULE), according to the selected variables.

## 3. Results

Estimates of total life expectancy at age 60 during the mid-2000s (see Table 2) show, as expected, that older women have longer TLE than men as gender differences range from 4.9 years in Spain to 3.4 years in Costa Rica. While women in the three countries have similar life expectancies, the TLE is close to 2 years higher among male Costa Ricans compared to Spanish and Chilean men.

**Table 2:** Total life expectancy, healthy life expectancy and unhealthy life expectancy at age 60 in years and the percentage of healthy years by gender for Chile, Costa Rica and Spain during the mid-2000s.

Country	Life Expectancy	Men	(95% CI)	Women	(95% CI)	% Healthy years	
						Men	Women
**Chile (2004–06)**	TLE 60	21.06	(19.62–22.36)	25.80^a^	(23.85–27.65)	88.70%	83.60%
	HLE 60	18.68	(17.34–19.89)	21.57^a^	(19.93–22.93)		
	ULE 60	2.38	(1.75–3.25)	4.23^a^	(3.30–5.44)		
**Costa Rica (2005–07**	TLE 60	22.85	(17.89–26.42)	26.24	(21.38–29.95)	80.04%	71.07%
	HLE 60	18.29	(14.19–21.07)	18.65	(15.20–21.21)		
	ULE 60	4.55	(3.1–6.57)	7.59	(5.04–10.44)		
**Spain (2004–07)**	TLE 60	21.04	(18.6–23.35)	25.97 ^b^	(23.07–28.89)	90.02%	81.13%
	HLE 60	18.94	(16.62–20.73)	21.07	(18.89–23.07)		
	ULE 60	2.10	(1.32–3.35)	4.89 ^a^	(3.30–6.89)		

Note: TLE: Total Life Expectancy; HLE: Healthy Life Expectancy; ULE: Unhealthy Life Expectancy. Values for women differ from men at

^a^p<0.05

^b^p<0.1.

LEs calculated with “msm” and “elect” R Packages, Confidence Intervals are computed from 500 replications. Estimations are based on the surveys EPS (Chile), CRELES (Costa Rica) and SHARE (Spain).

In each country, gender differences are also observed in unhealthy LE, with older women living more years in poor health than their male counterparts, especially in Spain and Costa Rica (about an additional 3 years). This also translates to a 5%-9% lower proportion of years that women live in good health. In terms of absolute values, country-differences in expected unhealthy years (ULE) at age 60 range from 7.6 years in Costa Rica to 4.2 years in Chile. Women in Costa Rica have a 3.3-years lower healthy life expectancy (HLE) and women in Spain a 0.6-year lower HLE than Chilean women.

The pattern is slightly different in the case of men. During the period studied, TLE at age 60 of Costa Ricans (22.8 years) was 1.8 years higher than what was estimated for both Spanish and Chilean men. On the other hand, levels of HLE were quite similar among the three countries (18.9, 18.7 and 18.7 years in, respectively, Spain, Chile and Costa Rica). This implies that the expected proportion that 60-year-olds can expect to live in good health in Costa Rica (80.0%) is below that of Spain (90.0%) and Chile (88.7%) and that the ULE is also highest in Costa Rica (4.6 years, against 2.4 years in Chile and 2.1 years in Spain).

**Fig 1:**
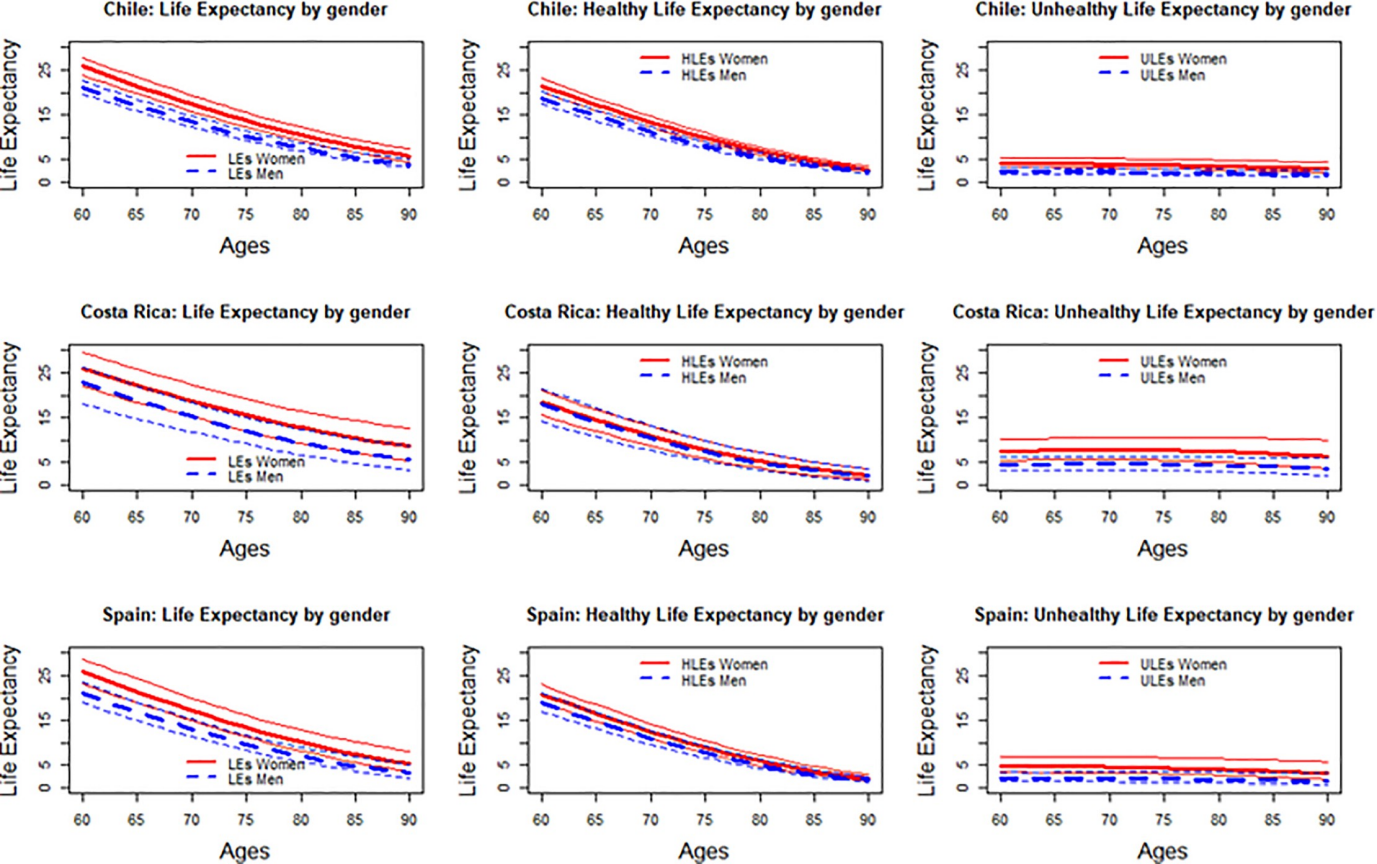
Total life expectancy, healthy life expectancy and unhealthy life expectancy from age 60 to 90, by sex. Chile, Costa Rica and Spain. Note: LEs calculated with “msm” and “elect” R Packages, Confidence Intervals computed from 500 replications.

If we analyse the TLE across ages, a monotonically downward trend for both sexes is observed from age 60 to 90 in the three countries (see Fig 1). This is consistent with a Gompertz distribution [68]. Regarding the trends for healthy LE, the country-specific trajectories converge from about age 80, whilst unhealthy LE remains remarkably constant across age. Both trends apply to each sex, although gender difference in unhealthy LE in Costa Rica and Spain are wider than in Chile. However, it should be noted that the sample of respondents aged 80+ is particularly small for Chile, making it difficult to estimate a robust pattern of life expectancy for this age group.

### Life expectancy by social participation

Our next step was to calculate marginal life expectancies according to different personal characteristics. First of all, estimates were made by educational level. Results showed no significant effect in either LE or HLE of older men and women in the three countries. However, in terms of the percentage of healthy years, the higher educated lived an estimated 1–2 percentage points more years in good health than the lower educated in the two LAC countries, 5 in the case of Spanish men and 8 percentage points in the case of Spanish women (see supplementary materials S4 Table and S4 Fig).

Larger differences in LE according to health status are observed for social participation, although none are statistically significant (Table 3). Social participation contributes to a higher TLE at age 60 in all three countries, with similar but non-significant differences observed among both sexes between those who participate socially and those who don’t, ranging between 1.0 years among Spanish men to 2.8 years among Costa Rican men.

**Table 3:** Total life expectancy, healthy life expectancy, unhealthy life expectancy and % healthy years at 60 years old by social participation and gender. Chile, Costa Rica and Spain.

Gender	Social Participation	Life Expectancies						% Healthy years		
		Chile (95% CI)		Costa Rica (95% CI)		Spain (95% CI)		Chile	Costa Rica	Spain
**Women**	TLE with participation	27.12	(24.11–30.91)	27.40	(21.93–32.01)	26.97	(23.24–30.84)			
	TLE without participation	25.34	(23.43–27.45)	25.09	(20.60–28.83)	25.75	(22.75–28.60)			
	HLE with participation	22.69	(20.41–25.34)	21.13	(16.81–24.70)	22.93	(19.77–26.09)	83.67%	77.12%	85.02%
	HLE without participation	21.07	(19.47–22.75)	16.69	(13.52–19.29)	20.55	(18.35–22.45)	83.15%	66.52%	79.81%
	ULE with participation	4.44	(2.71–6.87)	6.27	(3.94–9.41)	4.05	(2.33–6.84)			
	ULE without participation	4.27	(3.28–5.64)	8.40	(5.71–11.62)	5.19	(3.58–7.62)			
**Men**	TLE with participation	22.11	(19.61–24.95)	24.13	(17.24–28.86)	22.02	(17.26–26.09)			
	TLE without participation	20.75	(19.14–22.32)	21.31	(15.97–25.04)	20.98	(18.10–23.69)			
	HLE with participation	19.64	(17.4–21.78)	20.40	(14.38–24.52)	20.29	(16.03–23.81)	88.83%	84.54%	92.14%
	HLE without participation	18.35	(16.96–19.80)	16.35	(11.79–19.48)	18.64	(16.18–20.74)	88.43%	76.72%	88.85%
	ULE with participation	2.46	(1.36–4.36)	3.72	(2.14–6.14)	1.73	(0.85–3.46)			
	ULE without participation	2.40	(1.69–3.22)	4.95	(3.43–7.07)	2.34	(1.36–3.86)			

Note: TLE: Total Life Expectancy; HLE: Healthy Life Expectancy; ULE: Unhealthy Life Expectancy; a: Values are statistically different from older people who do not participate in formal social activities, p<0.05. Note: LEs calculated with “msm” and “elect” R Packages, Confidence Intervals computed from 500 replications. Estimations are based on EPS (Chile) data: 2004–2006. CRELES (Costa Rica) data: 2005–2007. SHARE (Spain) data: 2004–2007.

In the case of Costa Rica and Spain, the overall 1–3 years difference in TLE according to social participation is more than accounted for by extra healthy years as the socially active have roughly 4 additional healthy life years in Costa Rica and 2 extra years in Spain compared to those who are not active (levels are similar for men and women), while at the same time, ULE is between 0.6 (Spanish men) and 2.1 (Costa Rican women) years higher among those who do not partake in social participation. These HLE differences translate to a higher proportion of LE that is spend in good health among the socially active compared to those who don’t, especially in the case of Costa Rica (10% higher among women and 8% higher among men). Differences in Spain are, respectively, 5% and 3%. However, in absolute terms, the Spanish live more years in good health than Costa Ricans (up to 92% in the case of socially active men). Finally, in the case of Chile, virtually all of the additional 1.8/1.4 years of TLE among socially active women/men compared to the non-active is attributed to additional healthy years. As a result, there are no notable differences in the %HLE between the two social participation categories, although men have about a 5% advantage above women, with levels being most similar to those observed in Spain.

Lastly, even when we found no statistical differences between the dichotomous categories of education and social participation in the three LE indicators (see S5 Table), results showed that in terms of the proportion of life expectancy in good health (%HLE), there is a consistent educational gradient, especially in Spain (Table 4).

**Table 4:** Percentage of healthy years by social participation and educational level. Chile, Costa Rica and Spain.

Level of education and social participation	Women			Men		
	Chile	Costa Rica	Spain	Chile	Costa Rica	Spain
	Social participation: Yes					
**Educational level**						
Primary studies or lower	83.10	77.11	83.53	88.69	84.58	91.18
Secondary studies or higher	84.44	77.91	90.46	89.85	85.34	95.29
	**Social participation: No**					
Primary studies or lower	82.37	66.36	78.77	88.08	76.74	88.12
Secondary studies or higher	83.85	67.22	87.28	89.37	77.64	93.51

Note: Total and healthy life expectancies on which the percentage of healthy life years is based was calculated with “msm” and “elect” R Packages. Estimation are based on EPS (Chile) data (2004, 2006). CRELES (Costa Rica) data (2005, 2007) and SHARE (Spain) data (2004, 2007).

For instance, for secondary school or higher educated 60-year old Spanish men, 95% of their remaining life expectancy will be in good health if they also partake in social participation compared to 88% for those who were less educated. For the non-social participants, the percentages are, respectively, 1 and 3 percentage points lower. Although health of Spanish women is slightly worse than that of men, educational differences are about the same.

## 4. Discussion

This comparative study of old age people in Chile, Costa Rica and Spain, using three different panel data sources and multistate modelling for the estimation of marginal life expectancy and healthy years from age 60 onwards, provides new evidence on the relation between health and survival, taking into account gender, education and social participation.

The observation of women’s greater chance of survival but, at the same time, of having a higher number of unhealthy years than men is well known [6,48,51,76,77]. This “gender paradox” has been explained by a number of factors. While the female advantage in longevity is partly due to biological factors, women are also more likely to have suffered cumulative disadvantages during the life course because of socioeconomic and gender inequalities in educational attainment and employment conditions [78,79]. Additionally, there are specific behaviours and lifestyles differences, in particular obesity and medication use, where women perform worse than men [56,80,81]. Lastly, methodological issues like survey non-response and under-reporting of health problems by men may also be contributing factors [82].

This "gender paradox" is confirmed by our results, although with some exceptions. While all elderly women live longer but suffer a higher proportion of unhealthy years than men, for Costa Ricans there are no gender differences in HLE, although women, like in the other two countries, also live a greater proportion of years in an unhealthy state than men. In the particular case of Costa Rica, where the gender gap in LE was the smallest (3.4 years; see Fig 1), previous research that analysed causes of death has shown that the declining gender gap in LE is due to a more rapid reduction in male mortality, especially from circulatory system diseases [83].

Additionally, the fact that Costa Rica has an even higher male and female LE at age 60 than Spain, known to be one of the countries with the highest survival rates in the world [84], is not surprising given the existence of the so-called blue zones of exceptional longevity in Costa Rica [85,86]. In this sense, our study shows that Costa Rican men have a slight survival advantage over their peers in Chile and have longevity levels that are in the top ranking of longevity in the world, similar to Spain, Sweden, Canada, France, Japan and Switzerland but with a smaller gender gap [84,86,87]. Important known drivers of decreasing gender gaps in survival are changing patterns of risk factors, in particular the reduction in smoking among men [88]. On the other hand, the health condition of Costa Rican women is less favourable as they have the lowest percentages of healthy years compared to the other countries studied here and also compared to Mexico, Puerto Rico and the United States [89].

The advantage observed in Spain and Chile compared to Costa Rica in terms of healthy life years, especially regarding women, could be due to a cohort effect. Some studies suggest that the reduction in mortality and morbidity is largely attributable to changes in the early life cycle [90–92]. This is the case of the cohorts that benefited from the health improvements, economic progress and advances in health observed during the mid-1950s and especially after the post-war period in Spain. In addition, studies have shown that during the period 1985–2000, there was a disability compression in Spain, especially for women [93].

However, there are also other elements likely to play a role, including the type of diet and public expenditure on the national health system. With respect to the former, numerous studies suggest that the Mediterranean diet is an important factor in reducing morbidity and mortality for both functional capacities [94] and mental health [95]. In contrast, in Chile, even though, there are high rates of obesity, which is more frequent in women, affecting the difference in healthy years between men and women [96], a recent study conducted showed no evidence to support either a negative or a positive effect of overweight or obesity on LE and DFLE in Chile [56].

The continual improvements in life expectancy at all ages in each of the three countries over the last decades have also been attributed to the benefits of their health care systems, albeit that certain deficiencies do exist in the two LAC countries. While in Spain, health care has been universal and free of charge since 1986 [97], in Chile, it has had a mixed health system since the 1980s, composed of a public part called the National Health Fund (FONASA) and a private part, the Social Security Institutions (ISAPRES) [98]. Although most of the health coverage is assumed by the public part, the State invests relatively little in the health sector and the investment of public expenditure in the national health system is not sufficiently broad to cover the needs of the population in terms of primary and preferential health care, with the poor being the most disadvantaged [99].

In Costa Rica, although the health system has been universal and free of charge since the 1970s [100] and it is perceived by their population as efficient [101,102], there are substantial territorial inequalities in terms of morbidity and access to primary care [103]. The lower percentage of healthy years for women in Costa Rica, compared to the other two countries, may therefore be associated with geographical inequalities and differential access to health care.

In addition, the Costa Rican sample concentrates a higher proportion of cohorts born before 1930 (40%), against 27% and 36% of the sample in Chile and Spain. Especially younger cohorts were benefited by improved living conditions in the countries after the post-war period (1950-) (i.e. higher standards of hygiene, greater access to drinking water and health care, advances in medicine, economic development, educational expansion, healthier lifestyles, among others [26].

The minor variation in life expectancy by educational level in especially the Chilean and Costa Rican elders was also observed in Japan [104] and in older men in Mexico and supported by [105] and [106] who observed a greater contribution of material resources (such as income) than education to changes in health at older ages. The 5 and 8 percentage points more years in good health among higher compared to lower educated Spanish men and women is consistent with recent Spanish research that provided evidence of slowly increasing educational differences in life expectancy at age 50 since the 1960s [105].

### Diverse patterns by social participation

Results indicated differences in survival according to social participation, although not equally in each of the three countries analysed. Social participation increases LE and the percentage of healthy years in all three countries, but especially in Costa Rica, followed by Spain. The benefits of social participation in older adults are consistent with previous studies that indicate an increase in functional capacities, quality of life and well-being in terms of subjective health perception [107] and survival at older ages [41]. There is evidence that participation contributes to a lower mortality risk, although with differences by age and gender. Younger older women enjoy greater benefit from participation than their older male counterparts [108]. Activities associated with social participation such as volunteering (e.g. in elementary schools), participation in sports teams and clubs, increase strength, walking speed and general activity at follow-up [9] and lower future long-term care costs [37]. Additionally, such activities, that also include time spent with friends, are also reported to have a positive effect on survival in the older population [40].

In this sense, social participation has been proposed as a multi-purpose strategy to address different targets as psychological wellbeing, physical and cognitive functioning and interventions aimed to reduce health risk factors [107]. Regardless of the strategy implemented, participation provides the mechanisms for social support and social cohesion in the community so that older people can experience greater wellbeing within the framework of active ageing [109], reducing situations of dependency and the demand for care at very old ages. These benefits have been widely observed among older people in Costa Rica, due to initiatives promoted by the State and the National Council for Older Persons (CONAPAM) [110] and implemented by various government and non-profit institutions [111] to ease the economic and care burden on families, especially the most vulnerable groups.

However, considering the relatively low participation rates among older people in two of the country samples, ─Spain (23%) and Chile (32%)─ there appear to be barriers to social participation. The factors that explain participation rates among the elderly, particularly in the case of Chile, and the subsequent lack of significance of this indicator in their functional trajectories and survival, are related to their socio-economic and health conditions, but also the other way around [112]. For instance, people who have a functional limitation or a certain condition will have less capacity to participate actively in society due to that health condition.

Other known determinants of social participation in older people include the availability of individual (human capital, which includes educational level) and contextual resources (household composition, social networks, cultural factors) and the housing environment [107,113,114]. It is therefore important that effective strategies are developed and implemented that remove barriers, promote and facilitate social integration between local communities and their older population in order to preserve their functional capacity, both physically and cognitively, as well as their general wellbeing.

### Scope and limitations of the study

Although the analysed period roughly coincides between the three countries, one may consider the results somewhat outdated. However, we did this to ensure comparability, as otherwise a string of methodological issues would have placed a shadow on the comparability of the results. Nevertheless, in the supplementary material section we have included the results of a replication of the life expectancy estimations according to health status using all available longitudinal waves (Costa Rica: 2005, 2007, 2009; Chile: 2004, 2006, 2009, 2015, 2017; Spain 2004/05, 2007, 2011, 2013, 2015, 2017). The results do not significantly change for Spain. On the other hand, TLE at age 60 for Chile drops by almost 7 years between 2004–06 and 2009–15 in the case of men and 9 years in the case of women, with HLE dropping even more. The Costa Rican results for a later period also differs somewhat. Given these inconsistencies, we therefore decided to restrict our sample to only those who participated in the first two waves.

One important reason for this is the effect of attrition, i.e. the loss of individuals during survey follow-ups. This is a problem that is commonly reported in panel-type studies [115–118]. Using all wave samples would have left us with a final population that was not representative of the national population (see S1–S3 Figs). Attrition in terms of participating in panel survey is known to increase with age and, at least in the Chilean sample, is more likely to occur among people with a higher level of education [64].

On the other hand, as mortality is known to be higher in lower social strata, it changes the composition of the surviving population towards a selection of more “robust” individuals which, in turn, may weaken the observed relationship. This may explain why the lower educated in Chile and Costa Rica had slightly (but insignificantly) higher total life expectancies than the higher educated (see S4 Table). Although we controlled for age, there is also evidence that people with better health conditions are more likely to participate in panel surveys [119] and, indeed, in terms of HLE, educational differences were less (Chile) or reversed (Costa Rica).

Another issue that requires some clarification is that of proxy respondents. When it’s share of all respondents is considerable, as was the case of the first wave of the Costa Rican sample, it can have quite a distorting effect on the overall life expectancy. To illustrate: If we would have included the 661 cases who required a proxy respondent, the TLE at age 60 for women in the period 2005–2007 would have been 20.8 years and 20.0 years for men. By having taken these out of the calculations, the TLE increased to, respectively 26.2 and 22.9 years, i.e. levels that are similar to those obtained elsewhere (e.g. respectively 25.2 and 22.3 years in 2005 according to the WHO [6], see S7 Table). In turn, due to the highly selective initial Costa Rican sample that included a high number of proxies, the TLE for individuals who had a proxy respondent was just 13.5 years in the case of women and 12.5 years in the case of men. To be consistent, we also removed the 55 proxies used in the Spanish sample, while the Chilean sample did not use proxies.

A minor source of bias concerns the country comparison of the marginal life expectancy results according to social participation as they cover slightly different activities and items that are comparable but may have different wording (see S2 Table).

In the same vein, it should be mentioned that a different definition of limitation is likely to render different results. To facilitate comparison with other studies (see also next sub-section), we had measured health states according to a definition that had been developed for LAC countries who participated in the SABE study [120]. The authors identified an unhealthy state if the individual self-reported a limitation in at least one ADLs or two instrumental activities of daily living (IADL) or three types of mobility functional activities (MF) that measure the inability of the older adult to relate to his or her environment in daily life [45] (see also S2 Table). This definition had been used earlier by Moreno, Albala, Lera et al. [56].

However, due to the country differences in the phrasing and number of ADLs, IADLs and FM limitations (for instance, the Chilean survey only has one question on IADL and two on FM) it was more appropriate to adopt a more uniform definition of unhealthy state. We therefore opted to exclude the IADLs and FM limitations and define unhealthy state has having at least one out of four identical ADLs (eating/bathing/getting out the bed and walking across room). Hence, in order to get a sense of the sensitivity of these types of health indicators, we replicated the calculations of HLE for Costa Rica and Spain using the aforementioned definition and applying identical types of ADLs, IADLs and functional limitations (S8 Table). Results showed that particularly the health expectancy of women appear to be sensitive to the health indicator that is used as the overall years expected to live in good health is up to 5.6 years lower in the case of Costa Rican women according to the latter, broader, definition of functional limitation.

### Comparison with other studies

Studies report that the use of one or another indicator to measure health status also affects differences in healthy life years [121–123]. Even if the same indicator is used in a cross-national survey, variations in how the health question was translated in the respective questionnaires and country-specific perception of functional limitations may affect results [124,125]. Providing precise comparisons of our calculated healthy life years for Chile, Costa Rica and Spain with those from earlier studies are therefore hampered due to differences in methodology, indicators and sources. For instance, in the estimates on healthy years obtained by Moreno, Albala and Lera et al. [56] for Chilean older adults functional limitation was defined as having a limitation in at least one ADL, in two IADL, or in three mobility function activities, i.e. more restrictive than ours. Moreover, they also used a different longitudinal (panel) source (SABE) and one which only contained respondents from the city of Santiago. The initial sample was recruited in 1999–2000 and followed up in 2004–2005 and in 2009–2010. Their estimates of the proportion of remaining years from age 60 spent without functional limitation was about 25 percentage points lower than what we obtained (61.6% for men and 48.0% for women against our results of, respectively, 88.7% and 71.7%). On the other hand, if we would apply their indicator on the 2004–06 EPS dataset, we would obtain more similar results (65.3% and 62.6% for men and women, respectively).

Regarding Costa Rica there is more consistency between our results and those from other studies, including those by Rosero-Bixby [87] and Payne [89] that were based on the same CRELES survey. The first author estimated the total life expectancy for Costa Rica’s population aged 60, using a different methodology but results approximate those presented here as their estimates are one year below ours in the case of men two years in the case of women (men: 21.9 vs. 22.9 years and women 24.3 vs 26.2 years). The second study calculated LE according to health state from the age of 65 using "Multistate" models. Not being in a healthy state was defined as having at least one of 5 ADL limitations. Their results reported lower life expectancy values than those estimated in this study, but more similar than those reported by Rosero-Bixby (approximately one year less for both men and women). In terms of healthy years, his results report a fewer number of years in good health (12 healthy years for women and 14 years for men). This comparison with previous studies allows us to ascertain that in the case of Costa Rica the difference in the estimate of life expectancy is mainly due to the application of a different methodology as they used basically the same health indicator as we did here.

The results of healthy years and LE for Spain are very similar compared to those published in previous studies [79,105] and official statistics [6,126,127], despite the use of different methodologies and data sources for the calculation of LE. The other studies use health prevalence’s through the implementation of the Sullivan method [128–131] and do not consider changes in functionality over the age pattern, as it is possible when using incidence rates. Our study is therefore more robust as the current health and mortality dynamics on the life cycle at older ages are captured by the calculation of HLE and LE. Additionally, some of these other studies use health condition prevalence according to distinct health indicators in surveys [131–133] and the mortality experience from period life tables (which uses national death cases from vital registers and the whole population). Consequently, their results show a slightly lower proportion of healthy years than our estimates, while the life expectancies for both men and women are very similar. Lastly, as far as we are aware of, no study has yet looked at differences in HLE and LE in Spain according to social participation.

This observed difference between our results and those from previous research is mainly inherent to the source used [121,122]. In particular, mortality studies based on official mortality records have the entire population covered, including the institutionalized population, whereas our study only includes longitudinal information from a sample of private households. As a result, mortality from surveys can be at the same time underestimated, as the institutionalised population is normally excluded, or overestimated, when people who move from households to institutional care appear as deaths in surveys. Notwithstanding, overall, the mortality recorded in the surveys usually results in lower life expectancies than those estimated from vital records [134] as the population exposed to risk of mortality is underestimated due to effect of attrition in subsequent rounds of observation [135–137].

## Supporting information

S1 Fig Diagram showing longitudinal analysis of the EPS Data. Chile 2004–2017.
(DOCX)

S2 Fig Diagram showing longitudinal analysis of the CRELES Data. Costa Rica 2005–2009.
(DOCX)

S3 Fig Diagram showing longitudinal analysis of the SHARE Data. Spain 2004–2017.
(DOCX)

S4 Fig Male and female total life expectancy, healthy life expectancy and unhealthy life expectancy from 60 to 90, by educational level within countries. Chile, Costa Rica and Spain.
(DOCX)

S1 Table Results using all waves for Chile, Costa Rica and Spain: Total life expectancy, healthy life expectancy and the percentage of healthy years by gender. (DOCX)

S2 Table Questions of Activities of Daily Life (ADLs), Instrumental Activities of Daily Life (IADLs) and Mobility Function Activities (MF) by countries selected. (DOCX)

S3 Table Question of social participation by studied countries. (DOCX)

S4 Table Total life expectancy, healthy life expectancy and unhealthy life expectancy at 60 years old by educational level and gender. Chile, Costa Rica and Spain.
(DOCX)

S5 Table Total life expectancy, healthy life expectancy and unhealthy life expectancy at 60 years old and the percentage of healthy years by social participation and educational level. Chile, Costa Rica and Spain.
(DOCX)

S6 Table Percentage of healthy years by social participation and educational level. Chile, Costa Rica and Spain.
(DOCX)

S7 Table Comparison with official statistics and other studies. (DOCX)

S8 Table Comparison of results using an alternative definition of functional limitation. (DOCX)
