# Supplementary material for: The contribution of social participation to differences in life expectancy and healthy years among the older population: A comparison between Chile, Costa Rica and Spain
Source: PLoS One. 2021 Mar 12;16(3):e0248179. doi: 10.1371/journal.pone.0248179 (PMC7954322; doi:10.1371/journal.pone.0248179)
Supplement: S1 Fig — Chile 2004–2017. (DOCX) [file pone.0248179.s001.docx]

**S2 Fig. Diagram showing longitudinal analysis of the EPS Data. Chile 2004-2017**

Notes:

^1/^ Deaths reported between waves 2004 and 2006. From those, 68 death records didn’t identify exact date of death. Therefore, we´ve just analysed 186 deaths.

^2/^Deaths reported between waves 2006 and 2009. From those, 33 death records didn’t identify exact date of death. Therefore, we´ve just analysed 340 deaths.

^3/^Deaths reported between waves 2009 and 2015. They also included those reported in the wave 5 in 2012. From all death records (period 2009-2015), information of 159 deaths didn’t identify exact date of death. Therefore, we´ve just analysed 761 deaths.

^a/^Population entering to the group aged 60 and over in this wave. They were interviewed in previous waves but as they were under 60 years old they did not enter to the analysis population until this wave.

^b/^Additional to the population entering to the group aged 60 and over in this wave, these new cases also refer to new interviewees.
