# Supplementary material for: The contribution of social participation to differences in life expectancy and healthy years among the older population: A comparison between Chile, Costa Rica and Spain
Source: PLoS One. 2021 Mar 12;16(3):e0248179. doi: 10.1371/journal.pone.0248179 (PMC7954322; doi:10.1371/journal.pone.0248179)
Supplement: S3 Fig — Spain 2004–2017. (DOCX) [file pone.0248179.s003.docx]

**S4 Fig. Diagram showing longitudinal analysis of the SHARE Data. Spain 2004-2017**

Notes:

^1/^ Deaths reported between waves 2004 and 2007. From those, 6 death records didn’t identify exact date of death. Therefore, we´ve just analysed 85 deaths.

^2/^Deaths reported between waves 2007 and 2011. They also included those reported in the wave 3, 2009. From all death records (period 2007-2011), Information of 3 deaths didn’t identify exact date of death. Therefore, we´ve just analysed 262 deaths.

^3/^Deaths reported between waves 2011 and 2013. From those, 1 death records didn’t identify exact date of death. Therefore, we´ve just analysed 250 deaths.

^4/^Deaths reported between waves 2013 and 2015. From those, 15 death records didn’t identify exact date of death. Therefore, we´ve just analysed 387 deaths.

^5/^Deaths reported between waves 2015 and 2017. From those, 28 death records didnt identify exact date of death. Therefore, we´ve just analysed 380 deaths

^a/^ New cases refer to population entering to the group aged 60 and over in this wave (they were interviewed in previous waves but as they were under 60 years old they did not enter to the analysis population until this wave) and there are other people (60+) that refer to new interviewees in waves 2, 4, 5, 6 and 7.
