# Supplementary material for: The contribution of social participation to differences in life expectancy and healthy years among the older population: A comparison between Chile, Costa Rica and Spain
Source: PLoS One. 2021 Mar 12;16(3):e0248179. doi: 10.1371/journal.pone.0248179 (PMC7954322; doi:10.1371/journal.pone.0248179)
Supplement: S4 Fig — Chile, Costa Rica and Spain. (DOCX) [file pone.0248179.s004.docx]

**S8 Fig. A. Male Total Life Expectancy, Healthy Life Expectancy and Unhealthy Life Expectancy from 60 to 90, by educational level within countries. Chile, Costa Rica and Spain**

**
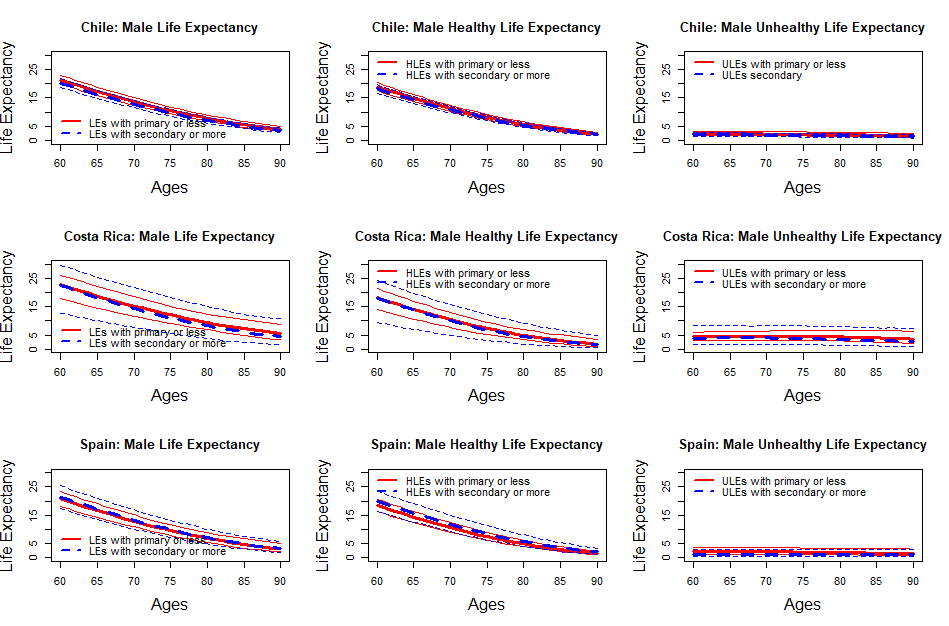
**

Note: TLE: Total Life Expectancy; HLE: Healthy Life Expectancy; ULE: Unhealthy Life Expectancy; LEs calculated with “msm” and “elect” R Packages, Confidence Intervals are computed from 500 replications. Estimations are based on EPS (Chile) data: 2004-2006. CRELES (Costa Rica) data: 2005-2007. SHARE (Spain) data: 2004-2007

**S8 Fig. B Female Total Life Expectancy, Healthy Life Expectancy and Unhealthy Life Expectancy from 60 to 90 by educational level within countries. Chile, Costa Rica and Spain**

**
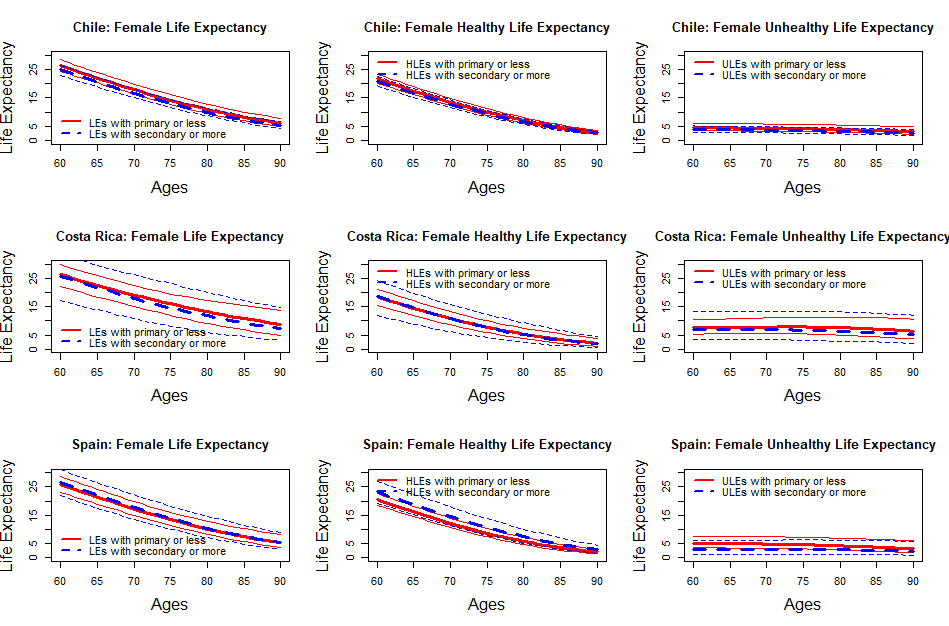
**

Note: TLE: Total Life Expectancy; HLE: Healthy Life Expectancy; ULE: Unhealthy Life Expectancy; LEs calculated with “msm” and “elect” R Packages, Confidence Intervals are computed from 500 replications. Estimations are based on EPS (Chile) data: 2004-2006. CRELES (Costa Rica) data: 2005-2007. SHARE (Spain) data: 2004-2007.
