# Supplementary material for: The contribution of social participation to differences in life expectancy and healthy years among the older population: A comparison between Chile, Costa Rica and Spain
Source: PLoS One. 2021 Mar 12;16(3):e0248179. doi: 10.1371/journal.pone.0248179 (PMC7954322; doi:10.1371/journal.pone.0248179)
Supplement: S1 Table — (DOCX) [file pone.0248179.s005.docx]

**S1 Table. Results using all waves for Chile, Costa Rica and Spain: Total Life Expectancy, Healthy Life Expectancy and the percentage of healthy years by gender.**

| **Country** | **Period** | **Men** | | **Women** | | | **Men** | | **Women** | | | **% Healthy years** | |
| --- | --- | --- | --- | --- | --- | --- | --- | --- | --- | --- | --- | --- | --- |
|  |  | **LE (95% CI)** | | **LE (95% CI)** | | | **HLE (95% CI)** | | **HLE (95% CI)** | | | **Men** | **Women** |
| **Costa Rica** | 2004-2007 | 22.85 | (17.89-26.42) | 26.24 |  | (21.38-29.95) | 18.29 | (14.19-21.07) | 18.65 |  | (15.2-21.21) | 80.0% | 71.1% |
|  | 2007-2009 | 27.41 | (20.12-32.37) | 25.35 |  | (18.26-29.48) | 15.12 | (10.47-19.01) | 12.64 |  | (18.26-29.48) | 55.2% | 49.9% |
| **Chile** | 2004-2006 | 21.06 | (19.62-22.36) | 25.80 | ^a^ | (23.85-27.65) | 18.68 | (17.34-19.89) | 21.57 | ^a^ | (19.93-22.93) | 88.7% | 83.6% |
|  | 2006-2009 | 16.95 | (15.06-18.69) | 23.43 | ^a^ | (20.85-25.53) | 13.02 | (10.99-15.13) | 17.53 | ^a^ | (15.13-19.76) | 76.8% | 74.8% |
|  | 2009-2015 | 14.50 | (12.4-16.49) | 16.95 |  | (14.64-19.03) | 7.86 | (5.61-10.58) | 8.71 |  | (6.38-11.22) | 54.2% | 51.4% |
|  | 2005-2017 | - |  | - |  |  | - |  | - |  |  |  |  |
| **Spain** | 2005-2007 | 21.04 | (18.60-23.35) | 25.97 | ^a^ | (23.07-28.89) | 18.94 | (16.62-20.73) | 21.07 |  | (18.89-23.07) | 90.0% | 81.1% |
|  | 2007-2011 | 20.22 | (18.38-21.76) | 24.01 | ^a^ | (22.1-25.92) | 18.59 | (16.82-20.17) | 19.23 |  | (17.61-20.87) | 91.9% | 80.1% |
|  | 2011-2013 | 21.01 | (17.69-23.11) | 24.27 | ^a^ | (20.94-26.81) | 16.83 | (13.23-19.14) | 15.97 |  | (12.44-18.38) | 80.1% | 65.8% |
|  | 2013-2015 | 20.36 | (17.92-22.11) | 26.04 | ^a^ | (24.09-27.76) | 16.89 | (14.35-19.00) | 20.29 |  | (18.25-21.92) | 83.0% | 77.9% |
|  | 2015-2017 | 20.10 | (16.75-21.94) | 24.18 |  | (21.39-26.1) | 16.63 | (12.93-18.86) | 18.14 |  | (15.2-20.25) | 82.7% | 75.0% |

Note: Definition in our study: an individual is considered to be unhealthy if he or she self-reported a limitation in four ADLs. For the specific limitations, see Table S5. ^a^: Values differ from older men, p<0.05. LEs calculated with “msm” and “elect” R Packages, 500 replications. Estimation are based on Chile (EPS): 2004,2006, 2009. CRELES, Costa Rica: 2005,2007, 2009. Spain (SHARE): 2004,2007, 2011, 2013, 2015, 2017.
