# Supplementary material for: The contribution of social participation to differences in life expectancy and healthy years among the older population: A comparison between Chile, Costa Rica and Spain
Source: PLoS One. 2021 Mar 12;16(3):e0248179. doi: 10.1371/journal.pone.0248179 (PMC7954322; doi:10.1371/journal.pone.0248179)
Supplement: S2 Table — (DOCX) [file pone.0248179.s006.docx]

**S5A Table. Questions of Activities of Daily Life (ADLs) by countries selected**

Note: [*original wording in Spanish*]. Items used in the definition of functional mobility used in this study (4ADLs). Items used in the definition of functional mobility by Moreno, Albala, Lera et al. (55) (a limitation in at least one ADLs, two IADLs or in three questions) used in this study in the sensitivity analysis described in the discussion.

**S5B Table. Questions of Instrumental of Activities of Daily Life (IADLs) by countries selected.**

Note: [*original wording in Spanish*]. Items used in the definition of functional mobility by Moreno, Albala, Lera et al. (55) (a limitation in at least one ADLs, two IADLs or in three questions) used in this study in the sensitivity analysis described in the discussion.

**S5C Table. Questions of Functional Mobility Activities by countries selected.**

Note: [*original wording in Spanish*]. Items used in the definition of functional mobility by Moreno, Albala, Lera et al. (55) (a limitation in at least one ADLs, two IADLs or in three questions) used in this study in the sensitivity analysis described in the discussion.
