# Supplementary material for: The contribution of social participation to differences in life expectancy and healthy years among the older population: A comparison between Chile, Costa Rica and Spain
Source: PLoS One. 2021 Mar 12;16(3):e0248179. doi: 10.1371/journal.pone.0248179 (PMC7954322; doi:10.1371/journal.pone.0248179)
Supplement: S3 Table — (DOCX) [file pone.0248179.s007.docx]

**S6 Table. Question on Social participation by studied countries**

Note: [*original wording in Spanish*]
