# Supplementary material for: The contribution of social participation to differences in life expectancy and healthy years among the older population: A comparison between Chile, Costa Rica and Spain
Source: PLoS One. 2021 Mar 12;16(3):e0248179. doi: 10.1371/journal.pone.0248179 (PMC7954322; doi:10.1371/journal.pone.0248179)
Supplement: S4 Table — Chile, Costa Rica and Spain. (DOCX) [file pone.0248179.s008.docx]

**S7 Table. Total Life Expectancy, Healthy Life Expectancy and Unhealthy Life Expectancy at 60 years old by educational level and gender. Chile, Costa Rica and Spain**

TLE: Total Life Expectancy; HLE: Healthy Life Expectancy; ULE: Unhealthy Life Expectancy; a: Values differ from elders with primary studies, p<0.05. LEs calculated with “msm” and “elect” R Packages, 500 replications.
