# Supplementary material for: The contribution of social participation to differences in life expectancy and healthy years among the older population: A comparison between Chile, Costa Rica and Spain
Source: PLoS One. 2021 Mar 12;16(3):e0248179. doi: 10.1371/journal.pone.0248179 (PMC7954322; doi:10.1371/journal.pone.0248179)
Supplement: S5 Table — Chile, Costa Rica and Spain. (DOCX) [file pone.0248179.s009.docx]

**S9 Table. Total Life Expectancy, Healthy Life Expectancy and Unhealthy Life Expectancy at 60 years old and the percentage of healthy years by Social Participation and educational level*.* Chile, Costa Rica and Spain.**

| **Gender** | **Type of life expectancy by level of education and social participation** | **Life Expectancies** | | | | | | **% Healthy years** | | |
| --- | --- | --- | --- | --- | --- | --- | --- | --- | --- | --- |
|  |  | **Chile (95% CI)** | | **Costa Rica (95% CI)** | | **Spain (95% CI)** | | **Chile** | **Costa Rica** | **Spain** |
| **Women** | TLE, Educ(P-) with participation | 27.52 | (24.31-30.98) | 27.48 | (22.22-32.21) | 27.14 | (22.62-31.13) |  | | |
|  | TLE, Educ(S+) with participation | 26.10 | (23.27-28.98) | 26.62 | (17.86-35.52) | 27.68 | (21.83-33.82) |  |  |  |
|  | TLE, Educ(P-) without participation | 25.92 | (23.83-28.07) | 25.27 | (20.49-28.95) | 25.76 | (22.91-29.17) |  |  |  |
|  | TLE, Educ(S+) without participation | 24.46 | (22.35-26.57) | 24.31 | (15.13-32.33) | 26.41 | (21.19-31.69) |  |  |  |
|  |  | | | | | | | | | |
|  | HLE, Educ(P-) with participation | 22.87 | (20.64-25.10) | 21.19 | (17.07-24.96) | 22.67 | (18.92-26.22) | 83.10% | 77.11% | 83.53% |
|  | HLE, Educ(S+) with participation | 22.04 | (19.69-24.84) | 20.74 | (13.18-26.83) | 25.04 | (19.87-29.90) | 84.44% | 77.91% | 90.46% |
|  | HLE, Educ(P-) without participation | 21.35 | (19.57-22.79) | 16.77 | (13.61-19.50) | 20.29 | (18.03-22.42) | 82.37% | 66.36% | 78.77% |
|  | HLE, Educ(S+) without participation | 20.51 | (18.47-22.48) | 16.34 | (9.79-22.25) | 23.05 | (18.92-27.44) | 83.85% | 67.22% | 87.28% |
|  |  | | | | | | | | | |
|  | ULE, Educ(P-) with participation | 4.65 | (2.79-7.37) | 6.29 | (3.68-9.71) | 4.47 | (2.40-7.56) |  | | |
|  | ULE, Educ(S+) with participation | 4.06 | (2.50-6.26) | 5.88 | (2.73-12.11) | 2.64 | (0.94-6.29) |  |  |  |
|  | ULE, Educ(P-) without participation | 4.57 | (3.44-6.07) | 8.5 | (5.56-11.72) | 5.46 | (3.72-8.17) |  |  |  |
|  | ULE, Educ(S+) without participation | 3.96 | (2.87-5.14) | 7.97 | (3.86-14.57) | 3.36 | (1.35-7.19) |  |  |  |
|  |  | | | | | | |  |  |  |
| **Men** | TLE, Educ(P-) with participation | 22.29 | (19.81-24.77) | 24.12 | (17.43-28.84) | 21.88 | (17.10-26) |  |  |  |
|  | TLE, Educ(S+) with participation | 21.28 | (18.65-23.83) | 23.26 | (12.39-30.46) | 22.30 | (16.65-27.14) |  |  |  |
|  | TLE, Educ(P-) without participation | 21.06 | (19.42-22.68) | 21.37 | (16.53-24.90) | 20.71 | (18.28-23.13) |  |  |  |
|  | TLE, Educ(S+) without participation | 20.03 | (18.43-21.64) | 20.48 | (11.86-27.74) | 21.25 | (16.81-25.85) |  |  |  |
|  |  | | | | | | | | | |
|  | HLE, Educ(P-) with participation | 19.77 | (17.82-21.59) | 20.4 | (14.24-24.61) | 19.95 | (15.86-23.81) | 88.69% | 84.58% | 91.18% |
|  | HLE, Educ(S+) with participation | 19.12 | (16.97-21.08) | 19.85 | (10.04-26.77) | 21.25 | (15.99-25.62) | 89.85% | 85.34% | 95.29% |
|  | HLE, Educ(P-) without participation | 18.55 | (17.25-20.00) | 16.4 | (12.27-19.26) | 18.25 | (15.95-20.31) | 88.08% | 76.74% | 88.12% |
|  | HLE, Educ(S+) without participation | 17.90 | (16.48-19.48) | 15.9 | (8.38-21.41) | 19.87 | (15.97-23.76) | 89.37% | 77.64% | 93.51% |
|  |  | | | | | | |  | | |
|  | ULE, Educ(P-) with participation | 2.52 | (1.49-3.89) | 3.72 | (1.99-6.29) | 1.92 | (0.91-3.63) |  | | |
|  | ULE, Educ(S+) with participation | 2.16 | (1.26-3.47) | 3.41 | (1.41-7.57) | 1.05 | (0.32-2.70) |  |  |  |
|  | ULE, Educ(P-) without participation | 2.51 | (1.79-3.33) | 4.98 | (3.20-7.01) | 2.46 | (1.45-3.86) |  |  |  |
|  | ULE, Educ(S+) without participation | 2.13 | (1.43-2.89) | 4.57 | (2.04-9.39) | 1.39 | (0.53-3.18) |  |  |  |

Note: TLE: Total Life Expectancy; HLE: Healthy Life Expectancy; ULE: Unhealthy Life Expectancy; P-: Primary studies or lower; S+: Secondary studies or higher; a: Values differ from elders with social participation within the same educational level, p<0.05. LEs calculated with “msm” and “elect” R Packages, Confidence Intervals are computed from 500 replications. Estimation are based on EPS (Chile) data: 2004-2006. CRELES (Costa Rica) data: 2005-2007. SHARE (Spain) data: 2004-2007
