# Supplementary material for: The contribution of social participation to differences in life expectancy and healthy years among the older population: A comparison between Chile, Costa Rica and Spain
Source: PLoS One. 2021 Mar 12;16(3):e0248179. doi: 10.1371/journal.pone.0248179 (PMC7954322; doi:10.1371/journal.pone.0248179)
Supplement: S6 Table — Chile, Costa Rica and Spain. (DOCX) [file pone.0248179.s010.docx]

**S10 Table. Percentage of healthy years by Social Participation and educational level*.* Chile, Costa Rica and Spain.**

Note: P-: Primary studies or lower; S+: Secondary studies or higher. LEs calculated with “msm” and “elect” R Packages. Estimation are based on EPS (Chile) data: 2004-2006. CRELES (Costa Rica) data: 2005-2007. SHARE (Spain) data: 2004-2007
