# Supplementary material for: The contribution of social participation to differences in life expectancy and healthy years among the older population: A comparison between Chile, Costa Rica and Spain
Source: PLoS One. 2021 Mar 12;16(3):e0248179. doi: 10.1371/journal.pone.0248179 (PMC7954322; doi:10.1371/journal.pone.0248179)
Supplement: S7 Table — (DOCX) [file pone.0248179.s011.docx]

**S11 Table. Comparison with Official Statistics and other studies**

| **Life Expectancy and Healthy Life Expectancy at 60** | | | | | | | |
| --- | --- | --- | --- | --- | --- | --- | --- |
| **Data Source** | **Index** | Chile | Costa Rica | Spain | Chile | Costa Rica | Spain |
|  |  |  | Women |  |  | Men |  |
| WHO (2005) | LE60 | 24.15 | 24.82 | 25.70 | 19.89 | 21.77 | 21.13 |
|  | HLE60 | 18.52 | 19.25 | 20.23 | 15.33 | 17.20 | 16.97 |
|  |  |  |  |  |  |  |  |
| EHLEIS (2006) | LE60 |  |  | 26.13 |  |  | 21.55 |
|  | HLE60 |  |  | 20.52 |  |  | 18.90 |
|  |  |  |  |  |  |  |  |
| Eurostat (2006) | LE60 |  |  | 26.42 |  |  | 21.67 |
|  | HLE60 |  |  | 20.67 |  |  | 18.98 |
|  |  |  |  |  |  |  |  |
| Moreno et al (2018) | LE60 | 20.4 |  |  | 16.4 |  |  |
| Santiago de Chile, Chile (2000-2009) | HLE60 | 9.9 |  |  | 10.2 |  |  |
|  |  |  |  |  |  |  |  |
| Rosero-Bixby (2018) | LE60 |  | 24.3 |  |  | 21.9 |  |
|  |  |  |  |  |  |  |  |
| Payne (2015) | LE65 |  | 20.2 |  |  | 19.5 |  |
|  | HLE65 |  | 12.5 |  |  | 14 |  |
|  |  |  |  |  |  |  |  |
| Our study | LE60 | 25.80 | 26.24 | 25.97 | 21.06 | 22.85 | 21.04 |
|  | HLE60 | 21.57 | 18.65 | 21.07 | 18.68 | 18.29 | 18.94 |

Notes: LE 60: Life Expectancy at 60 years old. HLE60: Healthy Life Expectancy at 60.

Studied periods in our study: Chile 2004-06; Costa Rica 2005-07; Spain 2004-07. For Chile, we used the more recent Longitudinal Social Protection Survey (EPS), while Moreno et al (2018) used a 10-year longitudinal sample from the Chilean cohort of the Study of Health, Ageing and Well-Being. However, their HLE was based on virtually the same health indicators as ours, reason why the HLE/LE proportion is very similar.

Rosero-bixby (2018) used the same longitudinal data from CRELES survey as in our study. He estimated Life expectancy by means of two-parameter Gompertz function and Monte Carlo simulation.

Payne (2015) also used CRELES data and HLE was based on ADLs indicators. His estimations are based on microsimulation to calculate the multistate life tables

Sources: WHO (2018); Global Health Observatory data repository. Available from: <http://apps.who.int/gho/data/node.main.688?lang=en>;

EUROSTAT (2020) Healthy life years and life expectancy at age 65 by sex. Available from: https://ec.europa.eu/eurostat/tgm/table.do?tab=table&plugin=1&language=en&pcode=tepsr_sp320; EHLEIS (2019) European Health & Life Expectancy Information System. Available from: <http://www.eurohex.eu/IS/web/app.php/Ehleis/Survey/Health?SubTyp=None>

Moreno X, Albala C, Lera L, Leyton B, Angel B, Sánchez H. (2018) Gender, nutritional status and disability-free life expectancy among older people in Santiago, Chile. PLoS One, Mar 28;13(3):18. Available from: <https://dx.plos.org/10.1371/journal.pone.0194074>.

Payne CF (2015) Aging in the Americas: Disability-free Life Expectancy Among Adults Aged 65 and Older in the United States, Costa Rica, Mexico, and Puerto Rico. Journals Gerontol Ser B Psychol Sci Soc Sci, Sep 7;73(2). Available from: <https://academic.oup.com/psychsocgerontology/article-lookup/doi/10.1093/geronb/gbv076>

Rosero-Bixby L (2018) High life expectancy and reversed socioeconomic gradients of elderly people in Mexico and Costa Rica. Demogr Res, Jan 5;38(1):95–108. Available from: https://www.demographic-research.org/volumes/vol38/3

| **Definitions, methodologies and data used by Official Statistics and other studies** | |
| --- | --- |
| Healthy life expectancy (HLE) is defined by the WHO as the Average number of years that a person can expect to live in "full health" by taking into account years lived in less than full health due to disease and/or injury. It adds up expectation of life for different health states, adjusted for severity distribution making it sensitive to changes over time or differences between countries in the severity distribution of health states. | |
| WHO Method of estimation: The equivalent lost healthy year fractions required for the HALE calculation are estimated as the all-cause years lost due to disability (YLD) rate per capita, adjusted for independent comorbidity, by age, sex and country. Sullivan's method uses the equivalent lost healthy year fraction (adjusted for comorbidity) at each age in the current population (for a given year) to divide the hypothetical years of life lived by a period life table at different ages into years of equivalent full health and equivalent lost healthy years (7)) | |
| EHLEIS: The data required to calculate Healthy Life Expectancy in European Countries are the age-specific prevalence (proportions) of the population in healthy and unhealthy states (often obtained from cross-sectional surveys), and age-specific mortality information taken from a period life table. Estimates are calculated following Sullivan’s Method. The results shown in the table are based on the ADL indicator from the SHARE survey (8) | |
| EUROSTAT: Estimates are calculated following Sullivan’s method (again applied to the life table) and the Healthy life years (HLY) indicator are provided by the GALI (Global Activity Limitation Index) question from EU-SILC | |
| **Mortality data used to calculate Life Expectancy by WHO vary between countries** (9): |  |
| Spain Life expectancy at birth: Based on official estimates of life expectancy available through 2014. The age pattern of mortality is based on (a) registered deaths by age and sex through 2012 and underlying population by age and sex, and (b) life tables through 2014 from the Human Mortality Database. VR 1951-2015. | |
|  |  |
|  |  |
| Chile Life expectancy at birth: Based on life tables derived from registered deaths, and population by age and sex from 1950 to 2013 adjusted for infant and child mortality. The number of deaths was adjusted using the growth-balance method, VR 1954-2014 | |
|  |  |
|  |  |
| Costa Rica Life expectancy at birth: Based on life tables derived from registered deaths, adjusted using the growth-balance method, and population by age and sex from 1950 to 2013 adjusted for infant and child mortality. VR 1956-2014 | |
|  |  |
