# Supplementary material for: The contribution of social participation to differences in life expectancy and healthy years among the older population: A comparison between Chile, Costa Rica and Spain
Source: PLoS One. 2021 Mar 12;16(3):e0248179. doi: 10.1371/journal.pone.0248179 (PMC7954322; doi:10.1371/journal.pone.0248179)
Supplement: S8 Table — (DOCX) [file pone.0248179.s012.docx]

**S12.-Table Comparison of results using an alternative definition of functional limitation (Albala et al, 2004)^1^ for Costa Rica and Spain with the definition used in this study^2^: Healthy Life Expectancy and the percentage of healthy years by gender and country.**

| **Country** | **Definition of unhealthy state** | **Men** | | **Women** | | **% Healthy years** | |
| --- | --- | --- | --- | --- | --- | --- | --- |
|  |  | **HLE (95% CI)** | | **HLE (95% CI)** | | **Men** | **Women** |
| **Costa Rica** | 1. Albala et al (2004) | 14.80 | (12.39-16.94) | 13.03 | (11.27-14.65) | 63.36% | 50.25% |
|  | 2. This study | 18.29 | (14.19-21.07) | 18.65 | (15.2-21.21) | 80.04% | 71.07% |
| **Spain** | 1. Albala et al (2004) | 17.55 | (15.4-19.64) | 17.11 | (14.97-18.84) | 82.20% | 65.26% |
|  | 2. This study | 18.94 | (16.62-20.73) | 21.07 | (18.89-23.07) | 90.02% | 81.13% |

Note: 1. Definition Albala et al (2004): an individual is considered to be unhealthy if he or she self-reported a limitation in at least one ADLs or two IADLs or in three questions on functional mobility; 2. Definition in our study: an individual is considered to be unhealthy if he or she self-reported a limitation in four ADLs. For the specific limitations, see Table S5. ^a^: Values differ from older men, p<0.05. LEs calculated with “msm” and “elect” R Packages, 500 replications. Estimation are based on Chile (EPS): 2004,2006. CRELES, Costa Rica: 2005,2007. Spain(SHARE): 2004,2007.

1. Albala C, Lera L, García C, Arroyo P, Marín P, Bunout D. Searching a Common Definition for Functional Limitation in Latin America. Gerontologist. 2004;44:550
